# Can MyotonPRO Be Used to Assess the Muscles Surrounding the Shoulder Joint in Patients Who Have Undergone Arthroscopic Rotator Cuff Repair (ARCR) and Reverse Shoulder Arthroplasty (RSA)? A Review of the Current Evidence

**DOI:** 10.3390/jcm15052039

**Published:** 2026-03-07

**Authors:** Agnieszka Tomczyk-Warunek, Bartosz Cukierman, Piotr Nalewaj, Marcin Krzysztof Waśko, Piotr Piech, Anna Winiarska, Tomasz Skrzypek, Magdalena Lis, Andrea Weronika Gieleta, Jaromir Jarecki

**Affiliations:** 1 Department of Traumatology, Orthopedics and Rehabilitation, Medical University of Lublin, Jaczewskiego 8, 20-954 Lublin, Poland; agnieszka.tomczyk-warunek@umlub.edu.pl (A.T.-W.); piotr.nalewaj@umlub.edu.pl (P.N.); piotr.piech@umlub.edu.pl (P.P.); jaromir.jarecki@umlub.edu.pl (J.J.); 2 Department of Trauma and Orthopedic Surgery, Independent Public Health Care Facility in Sokółka, St. Gen. Wł. Sikorskiego 40, 16-100 Sokółka, Poland; bartoszcukierman@gmail.com; 3 Department of Radiology and Imaging, The Medical Centre of Postgraduate Education, 01-813 Warsaw, Poland; marcin@wasko.md; 4 Department of Correct, Clinical and Imaging Anatomy, Medical University of Lublin, 20-090 Lublin, Poland; 5 Department of Bromatology and Nutrition Physiology, Institute of Animal Nutrition and Bromatology, University of Life Sciences in Lublin, Akademicka 13, 20-950 Lublin, Poland; anna.mieczan@up.edu.pl; 6 Department of Biomedicine and Environmental Research, Faculty of Medicine, John Paul II Catholic University of Lublin, 20-708 Lublin, Poland; magdalena.lis@kul.pl; 7 Faculty of Medicine and Surgery, University of Malta, Mater Dei Hospital Tal Qroqq, Block A, 2090 Msida, Malta; andrea-weronika.gieleta@gov.mt

**Keywords:** arthroscopic rotator cuff repair (ARCR), reverse shoulder arthroplasty (RSA), biomechanical properties, viscoelastic properties, MyotonPRO, shoulder joint

## Abstract

**Background/Objectives**: Arthroscopic rotator cuff repair (ARCR) and reverse shoulder arthroplasty (RSA) are among the most commonly used surgical treatment methods. A growing number of studies assess the changes in the biomechanical and viscoelastic properties of the muscles and tendons surrounding the shoulder joint. Therefore, the aim of this study was to review the literature to determine how the biomechanical properties of the muscles surrounding the shoulder joint change as a result of ARCR and RSA, and whether MyotonPRO was used in this group of patients. **Methods**: A review of the international scientific literature was conducted in September 2025. The study was based on searches of the following databases: Google Scholar, PubMed, Scopus, and Web of Science. A final total of 32 articles were included in the review. **Results**: In this article, we have shown that ARCR and RSA procedures cause changes in the biomechanical properties of the muscles surrounding the shoulder joint. We also demonstrated that MyotonPRO has been used in this group of patients in a limited number of studies. However, the studies confirm that it is a reliable tool for examining upper limb muscles. **Conclusions**: This literature review demonstrates a new direction in research using MyotonPRO. Using this device in muscle testing in patients after ARCR and RSA will allow for a better understanding of the changes that occur in muscles as a result of these procedures, as research in this area is new and incomplete.

## 1. Introduction

**Figure 1:**
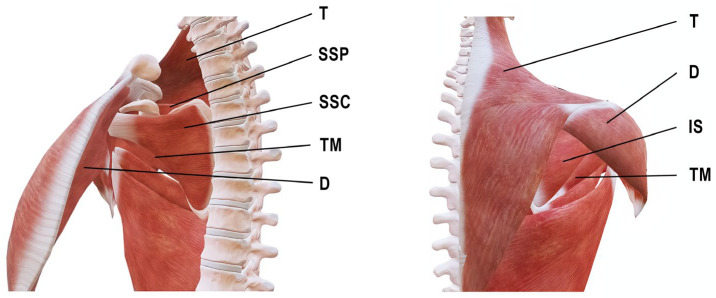
A graphical presentation the most important muscles acting on the shoulder joint. Legend: supraspinatus (SSP); infraspinatus (IS); teres minor (TM); and subscapularis (SSC); deltoid (D); pectoralis major (PM); and trapezius (T) [5].

The most important muscles acting on the shoulder joint are the rotator cuff muscles, which include the supraspinatus (SSP), infraspinatus (IS), teres minor (TM), and subscapularis (SSC). This muscle group enables a wide range of motion while simultaneously ensuring joint stability [1]. Other muscles that play an important role in normal shoulder function include the deltoid (D), pectoralis major (PM), and trapezius (T). The D is responsible for stabilization and motion of the shoulder joint and is divided into three parts: anterior (AD), middle (lateral) (MD), and posterior (PD). The AD cooperates with the PM and enables shoulder flexion during gait, whereas the PD, together with the latissimus dorsi (LaD), enables extension of the upper limb during gait [2]. The PM plays a key role in shoulder rotation, adduction, and flexion [3]. The T is divided into three parts: upper (UT), middle (MT), and lower (LT), and is responsible for scapular motion and stabilization, which significantly influences shoulder joint movement [4] (Figure 1).

Among the most frequently performed surgical procedures for shoulder joint disorders are arthroscopic rotator cuff repair (ARCR) and reverse shoulder arthroplasty (RSA) [6,7]. ARCR is performed in patients with rotator cuff tears, which constitute one of the most common injuries of the shoulder girdle [7], and represents an alternative to total shoulder arthroplasty [8]. In contrast, RSA is performed in patients after shoulder trauma and in individuals with rotator cuff arthropathy. RSA is also indicated in patients suffering from degenerative disease of the shoulder joint [6,9].

As a result of RSA, the anatomical structure of the joint changes, resulting in a change in the biomechanics of this joint, and thus, significantly affecting the muscles surrounding this joint [10,11,12]. It has been shown that increased activity and tension of this muscle occur as a result of RSA [13,14,15,16,17]. On the other hand, some studies did not demonstrate these changes or observed a decrease in the activity of this muscle, which may be related both to the variability of the studied patient populations and the technique of testing the biomechanical properties of muscles [16,18,19,20,21,22,23]. The number of studies examining other muscles of the rotator cuff and those important for the function of this joint is also limited [19,22,23]. In patients after ARCR, the SST and SSP muscles were most frequently examined, as this tendon is the most frequently torn in these patients [24]. However, there is little information on how SST tear and repair following ARCR affect the biomechanics of the remaining muscles surrounding this joint [25,26,27,28,29]. What is particularly important is that the studies examining patients after shoulder joint surgery are limited in number and are characterized by high variability in the examined patient population as well as the examination technique itself. The available literature includes only limited studies in which muscles were assessed using EMG (electromyography) or elastography after ARCR and RSA [14,15,16,17,18,25,26,27,28,29,30,31]. However, there is a lack of such studies using the MyotonPRO device (Myoton AS, Talinn, Estopnia).

Recently, MyotonPRO has been increasingly used in studies assessing the biomechanical and viscoelastic properties of muscles. The reliability of stiffness and elasticity measurements obtained with this device has been confirmed in numerous clinical studies involving both healthy individuals and patients with various neurological and musculoskeletal disorders, such as Parkinson’s disease, paratonia, cerebral palsy (MPDZ), and stroke [32,33,34,35,36,37,38,39,40]. The measurement of viscoelastic properties is also characterized by very high repeatability and reliability [35]. Myotonometry is successfully used in sports, medicine, and physiotherapy [32].

Compared with other methods used to assess the biomechanical properties of muscles and tendons, such as elastography and surface electromyography, MyotonPRO is less expensive and is practical, and non-invasive. It does not require specialized laboratory conditions, and the examination is easy to perform, requiring only appropriate training and the selection of correct anatomical landmarks for measurements [41].

It should be emphasized that each of these techniques has its limitations. Similar to elastography and EMG, MyotonPRO measurements are performed transcutaneously. Therefore, when interpreting myotonometric results, the properties of the skin and subcutaneous tissue should be taken into account. The biomechanical properties of muscles are also significantly influenced by their structure and muscle fiber architecture. These properties are additionally dependent on sex and age; therefore, studies using MyotonPRO should focus on the most homogeneous patient groups possible, and the examination should be supplemented with imaging results of the assessed muscle [32].

It should also be noted that studies using this device are relatively new, and there is still a lack of information regarding how stiffness, elasticity, and viscoelastic properties change in different patient populations. The available literature does not provide sufficient data on how muscle tissue properties change after RSA and ARCR. Although some studies indicate that stiffness and elasticity of the evaluated muscles change after these procedures, such studies are scarce and are usually conducted in small patient groups. Moreover, they do not assess the viscoelastic properties of the muscles [13,14,15,16,17,18,25,26,27,28,29,30,31].

Therefore, the primary aim of this study is to review the available literature regarding changes in the biomechanical and viscoelastic properties of the muscles surrounding the shoulder joint following RSA and ARCR. A secondary aim is to determine whether MyotonPRO has been used to assess these muscles.

## 2. Materials and Methods

### 2.1. Literature Search Methodology

The review was conducted in accordance with the Preferred Reporting Items for Systematic Reviews and Meta-Analyses extension for Scoping Reviews (PRISMA-ScR) (Supplementary File S1). The literature search was initially performed in July 2025 and updated in September 2025 (last search date: 30 September 2025). Two reviewers with clinical expertise in orthopedics and rehabilitation independently performed the literature search and study selection. Any disagreements were resolved by discussion; when consensus could not be reached, a third reviewer adjudicated.

Electronic searches were performed in PubMed/MEDLINE, Scopus, and Web of Science. In addition, Google Scholar was searched to identify the potentially relevant gray literature and recently indexed records. Search terms covered (i) the index test (MyotonPRO/myotonometry), (ii) the anatomical region and target muscles (shoulder/rotator cuff and surrounding muscles), and (iii) the clinical context and/or comparator assessment methods (ARCR/RSA, elastography, and EMG).

The PubMed strategy was developed first and then adapted for the remaining databases by modifying field tags and syntax as appropriate. PubMed (MEDLINE) example search strategy (last run: 30 September 2025): ((“MyotonPRO” [Title/Abstract] OR myotonometry [Title/Abstract] OR myotonometer* [Title/Abstract])) AND (shoulder [Title/Abstract] OR “rotator cuff” [Title/Abstract] OR supraspinatus [Title/Abstract] OR infraspinatus [Title/Abstract] OR subscapularis [Title/Abstract] OR “teres minor” [Title/Abstract] OR deltoid [Title/Abstract] OR trapezius [Title/Abstract] OR “pectoralis major” [Title/Abstract]) AND (“arthroscopic rotator cuff repair” [Title/Abstract] OR ARCR [Title/Abstract] OR “reverse shoulder arthroplasty” [Title/Abstract] OR RSA [Title/Abstract] OR (elastography [Title/Abstract] OR “shear wave elastography” [Title/Abstract] OR SWE [Title/Abstract] OR electromyograph* [Title/Abstract] OR EMG [Title/Abstract] OR stiffness [Title/Abstract] OR elasticity [Title/Abstract] OR viscoelastic* [Title/Abstract] OR biomechanical [Title/Abstract])) Filters applied: Humans, English, publication dates 1 January 2012–30 September 2025. For Google Scholar, results were sorted by relevance and the first 200 records were screened. To increase completeness, reference lists of included studies and relevant reviews were hand-searched. No automation tools were used at any stage of screening or selection.

### 2.2. Eligibility Criteria

#### 2.2.1. Inclusion Criteria

Studies were eligible for inclusion if they met all of the following criteria:

Population: adults (≥18 years), patients undergoing or having undergone arthroscopic rotator cuff repair (ARCR) or reverse shoulder arthroplasty (RSA) (direct evidence), or human participants in whom shoulder-region muscles (rotator cuff or shoulder girdle muscles) were assessed using MyotonPRO (indirect evidence supporting feasibility and reliability).

Intervention/Assessment Method: studies assessing biomechanical or viscoelastic muscle properties using MyotonPRO, elastography (including SWE or strain elastography), or electromyography (EMG).

Anatomical focus: supraspinatus, infraspinatus, subscapularis, teres minor, deltoid (anterior/middle/posterior), trapezius (upper/middle/lower), or pectoralis major, and/or their tendons.

Study design: clinical studies (prospective, retrospective, case–control, cohort studies).

Language and publication period: articles published in English between 1 January 2012 and 30 September 2025. The 2012 cut-off was selected because earlier studies primarily used previous generations of myotonometers (e.g., Myoton-2, Myoton-3), whereas this review focused on evidence related to MyotonPRO.

#### 2.2.2. Exclusion Criteria

Studies were excluded if they (a) included pediatric populations (<18 years of age), (b) assessed upper limb muscles other than those relevant to shoulder function, (c) used myometers other than MyotonPRO when Myoton-based results were the primary focus, (d) were case reports, conference abstracts without full text, narrative commentaries, or non-peer-reviewed publications, (e) were not available in English. Clarification of Scope:

Because only a very limited number of studies have evaluated MyotonPRO in patients after ARCR or RSA, studies investigating shoulder-region muscles in other clinical contexts (e.g., musculoskeletal or neurological conditions) or in healthy populations were included to provide indirect evidence regarding the feasibility, reliability, and potential applicability of MyotonPRO in this anatomical region.

These studies were analyzed separately from direct postoperative ARCR/RSA evidence.

### 2.3. Study Selection Process

All records identified through database searching were exported and duplicates were removed prior to screening. The screening process was conducted in two stages.

Stage 1—Title and abstract screening: two independent reviewers screened titles and abstracts for relevance according to the predefined eligibility criteria. Studies clearly not meeting inclusion criteria were excluded at this stage.

Stage 2—Full-text review: full texts of potentially eligible articles were retrieved and independently assessed by the same two reviewers. Reasons for exclusion at the full-text stage included (a) wrong population (e.g., pediatric or non-shoulder-related muscles), (b) wrong intervention/assessment method, (c) use of myometers other than MyotonPRO (when Myoton-based assessment was the focus), (d) non-clinical study design, (e) non-English publication.

Any discrepancies between reviewers at any stage of screening were resolved through discussion. If consensus could not be reached, a third reviewer made the final decision. The level of agreement between reviewers during title/abstract and full-text screening was assessed qualitatively through consensus discussion.

No automation tools or artificial intelligence-assisted screening software were used.

**Figure 2:**
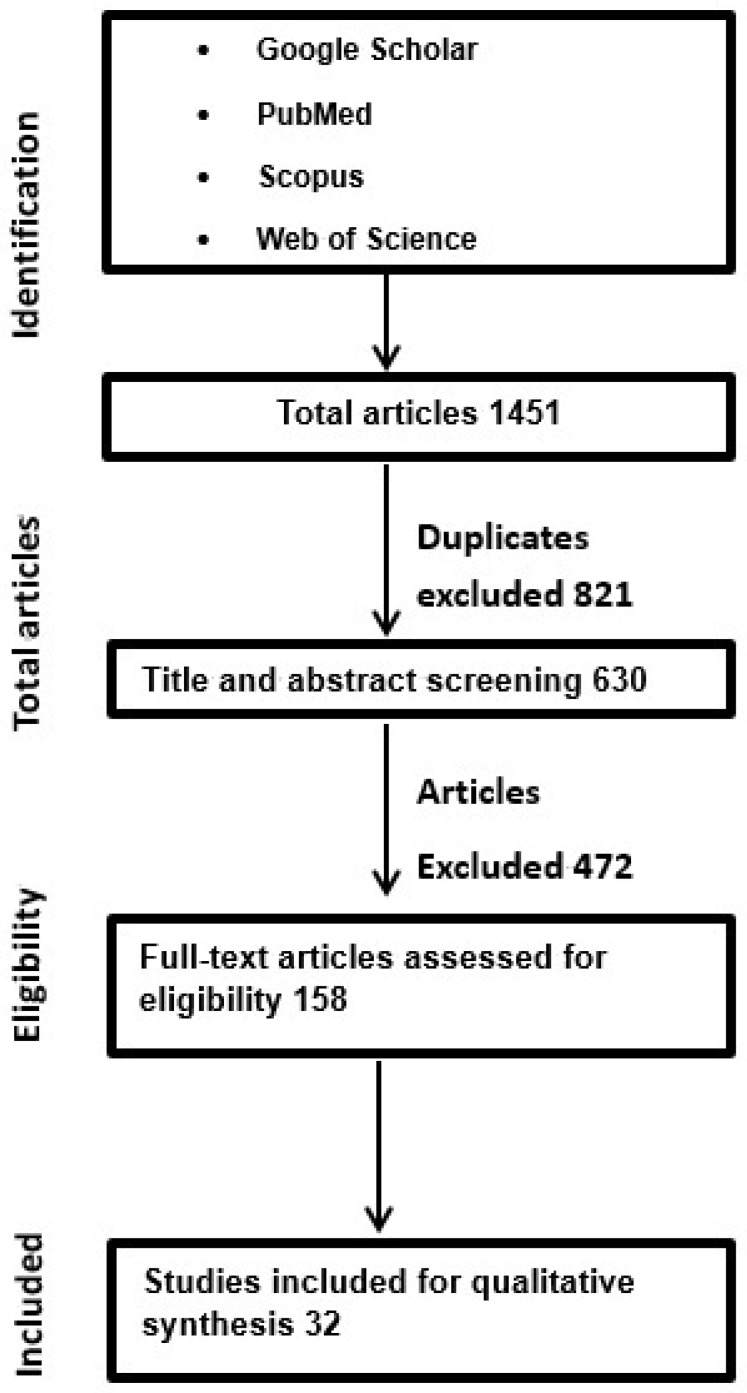
Presentation of how the literature review was conducted.

The study selection process is summarized in the PRISMA-ScR flow diagram (Figure 2).

### 2.4. Critical Appraisal of Included Studies

As this review was conducted as a scoping review in accordance with PRISMA-ScR guidelines, a formal risk-of-bias or methodological quality assessment was not performed. The objective of this review was to map the available evidence regarding (i) changes in biomechanical properties of shoulder-region muscles following ARCR and RSA and (ii) the use of MyotonPRO in this clinical context, rather than to quantitatively synthesize outcomes or compare intervention effectiveness. However, key methodological characteristics of the included studies—such as study design, sample size, population heterogeneity, follow-up duration, and assessment methods—were extracted and are described in the Section 3 and Section 4 to provide context regarding the strength and limitations of the available evidence.

## 3. Results

### 3.1. Elastography of the Shoulder Joint After Surgical Interventions—RSA and ARCR

It should be noted that the two main types of elastography are currently distinguished: static elastography and dynamic elastography. Static elastography, also referred to as strain elastography (SE), is based on the analysis of tissue deformations recorded by ultrasound, which are induced by compression with the ultrasound probe [42]. In contrast, dynamic elastography utilizes shear wave propagation and is based on measuring the velocity of these waves (shear wave speed, SWS) [43]. At present, dynamic elastography (shear wave elastography, SWE) is the most commonly used technique in musculoskeletal research.

#### 3.1.1. RSA—Reverse Shoulder Arthroplasty

##### Deltoid Muscle

In an in vivo SWE study, Schmalzl et al. (2022) [13] demonstrated that RSA leads to changes in the biomechanical properties of the D muscle, particularly in the anterior (AD) and middle (MD) portions. The study also showed that increased AD muscle tension was correlated with higher pain intensity following surgery [13].

Similarly, a study by Fenwick et al. (2023) [14] using elastography also demonstrated postoperative changes in D biomechanics following RSA. In this study, the operated limb was compared with the contralateral side, and significantly increased D stiffness was observed after surgery, especially in the AD and MD regions. However, these findings were not correlated with functional shoulder assessment or radiological evaluation of glenoid inclination and retroversion, which may be related to the small sample size (n = 18) and the high variability within the study group [14] (Table 1).

Alterations in D biomechanics were also reported in studies by Dukan et al. (2022) [15] and Hatta et al. (2024) [44]. The study by Dukan et al. (2022) [15] demonstrated an increase in SWS at rest and no significant differences in D stiffness during abduction when compared with healthy controls. Likewise, Hatta et al. (2024) [44] reported increased D stiffness and analyzed its postoperative time course. A negative correlation was observed between the increase in D stiffness measured at 3, 6, and 9 months postoperatively and the change in muscle strength recorded 3 months later [44] (Table 1).

In the study by Fischer et al. (2017) [16], ARFI elastography using acoustic radiation force impulse was applied. This investigation demonstrated greater D stiffness in patients after RSA compared with the contralateral side, accompanied by a simultaneous decrease in D elasticity on the operated side [16] (Table 1).

#### 3.1.2. ARCR—Arthroscopic Rotator Cuff Repair

##### Supraspinatus Tendon

In the clinical study conducted by Kim et al. (2023) [25], strain elastography (SE) was used to assess the elasticity of the reconstructed supraspinatus tendon (SST). The results demonstrated a significant correlation between SST elasticity and postoperative shoulder function and muscle strength. The findings indicated that patients after ARCR who exhibited stiffer regenerated SST also demonstrated greater muscle strength [25] (Table 1).

In the study by Hackett et al. (2023) [46], it was shown that SST stiffness did not change during the first 12 weeks after surgery across all examined tendon regions. However, within 6 months after ARCR, a 21% increase in stiffness was observed in the lateral portion of the tendon. Similar findings were reported for the medial portion located 3 mm from the footprint. In contrast, in the medial portion located 6 mm from the footprint, the increase in stiffness occurred 6 weeks later. A positive correlation between SST thickness and its stiffness was also demonstrated [46] (Table 1).

An increase in SST stiffness at 6 and 12 months following ARCR was also reported by Solari et al. (2024) [47]. In this study, a positive correlation between SST stiffness and SSP stiffness was additionally observed. Similarly, Itoigawa et al. (2020) [27] investigated SST and demonstrated that in patients with small or partial tears, tendon stiffness was higher at 1 week postoperatively compared with 3, 4, and 6 months after surgery. In contrast, in patients with medium and large tears, the SWE value was higher in the first postoperative week only in comparison with 3 and 6 months after surgery [27] (Table 1).

Conversely, in a study by Chen et al. (2024) [45], in which results were compared with a healthy control group, SST on the operated side before surgery was characterized by a lower shear wave velocity (SWV) value compared with the control group. Moreover, a negative correlation was demonstrated between preoperative SWV and ARCR outcomes, indicating that preoperative tendon stiffness may influence surgical results [45] (Table 1).

##### Supraspinatus and Infraspinatus Muscles

In the study by Sakaki et al. (2022) [26], SSP stiffness active was assessed in patients with rotator cuff tears (RCT) who were qualified for ARCR. The examined muscle was divided into four distinct regions based on muscle fiber arrangement: posterior superficial (PS), posterior deep (PD), anterior superficial (AS), and anterior deep (AD). The results demonstrated a reduction in active stiffness of the AS region of the SSP compared with other examined regions [26] (Table 1).

Similarly, Itoigawa et al. (2020) [27] evaluated SSP stiffness in the same four regions (AS, AD, PS, and PD) at the following time points: preoperatively and postoperatively at 7 days, and 1, 2, 3, 4, 5, and 6 months. In patients with small or partial rotator cuff tears, SWE values of the PD region of the SSP were significantly higher preoperatively compared with 7 days and 1 and 2 months after surgery. Stiffness of this region began to increase after 4 months and by the end of the observation period reached a value close to the preoperative level. In the AD region of the SSP in this group, stiffness was also higher preoperatively compared with measurements obtained at 7 days and 1, 2, and 3 months after ARCR [27] (Table 1).

In patients with medium- and large rotator cuff tears, PD region stiffness was lower at 1 month postoperatively compared with preoperative values and with measurements obtained at 4, 5, and 6 months after ARCR. In these patients, SWE values of the AD SSP region were lower at 1 month postoperatively compared with 7 days as well as 4 and 6 months after surgery. The study demonstrated that SSP stiffness changes over time during the healing process after ARCR and is dependent on the severity of the rotator cuff tear [27] (Table 1).

In the study by Jeong et al. (2022) [48], the relationship between preoperative SSP stiffness and elasticity and ARCR outcomes was evaluated. The authors demonstrated that higher preoperative SWE values were observed in patients with insufficient repair. Moreover, SWE-based elasticity measurements allowed identification of patients with inadequate repair independently of MRI measurements and assessment of fatty infiltration [48] (Table 1).

Similarly, in the study by Chen et al. (2024) [45], statistical analysis revealed that preoperative SSP stiffness was negatively correlated with postoperative ARCR outcomes. In addition, SSP stiffness on the operated side was lower compared with the control group [45] (Table 1).

Finally, in the study by Huang et al. (2022) [28], the most important conclusion was that SWE-measured stiffness of the rotator cuff muscles (IS and SSP) was significantly correlated with both the severity and size of the tear. Moreover, the elastic modulus values of the IS and SSP predicted the reparability of the rotator cuff. The results indicated that greater rotator cuff stiffness was associated with worse imaging outcomes, including muscle hypotrophy, increased fatty infiltration, and tendon retraction [28] (Table 1).

##### Deltoid Muscle

In studies evaluating the stiffness of muscles surrounding the shoulder joint after ARCR, the D muscle has also been assessed. These studies demonstrated that D stiffness changes over time following surgery. In the study by Hackett et al. (2023) [46], it was shown that D stiffness did not change up to 12 weeks after the procedure. However, between 1 and 52 weeks after ARCR, its stiffness increased by 15% [46]. In another study, it was demonstrated that D stiffness was significantly greater at 54 weeks after surgery compared with values at 1 and 12 weeks after ARCR [47] (Table 1).

### 3.2. Electromyography of the Shoulder Joint After Surgical Interventions—RSA and ARCR

Electromyography (EMG) is a technique used to assess the electrical activity of muscles. EMG detects action potentials generated by motor units within muscles. When an action potential is generated, depolarization occurs at the neuromuscular junction, leading to activation of muscle fiber contraction. EMG detects and records these changes [49].

This examination is performed using electrodes, which can be divided into two types: surface (non-invasive) electrodes—sEMG, and needle (invasive) electrodes—iEMG. Although needle EMG allows for more precise acquisition of motor unit activation signals because the electrode is inserted directly into the muscle, in clinical practice the surface method is more commonly used and preferred. This is due to easier electrode placement and minimal discomfort for the patient during the examination [50].

#### 3.2.1. RSA—Reverse Shoulder Arthroplasty

In studies involving patients after RSA, muscle activity has most frequently been assessed using sEMG [18,19,20,21,22,23]. Only in the studies by Fischer et al. (2017) [16] and Khazzam et al. (2020) [51] was iEMG used (Table 2).

##### sEMG—Deltoid Muscle

In the study by Pegreffi et al. (2017) [18], which included a two-year follow-up of patients after RSA, a lower D muscle activity on the operated side was observed in all three analyzed movements. In particular, significantly lower electromyographic activity of the anterior (AD) and middle (MD) portions was demonstrated in comparison with the non-operated side [18] (Table 2).

Similarly, in the study by Reinmuller et al. (2020) [20], which also included a two-year follow-up after RSA and compared the operated and non-operated sides, a reduction in antagonistic EMG activity of the latissimus dorsi (LD) on the operated side during flexion was observed. For the posterior deltoid (PD), antagonistic EMG activity was lower during extension and external rotation. In the case of the anterior deltoid (AD), lower neuromuscular activity during internal rotation was demonstrated. At the same time, this study also showed an increase in antagonistic EMG co-activation of the AD during external rotation [20] (Table 2).

In contrast, in the study by Pelletier-Roy et al. (2021) [22], results obtained in patients after RSA were compared with a healthy control group, and similar findings for the AD were reported. It was observed that during flexion, AD activity was lower in the RSA group compared with the control group. However, for the MD and PD, sEMG demonstrated a slight increase in their activity in the postoperative group. An increase in PD activity was also observed in the postoperative group during external rotation compared with the control group [22] (Table 2).

On the other hand, in the study by Pietroski et al. (2025) [19], in which sEMG was assessed at different time points after RSA, different results were demonstrated. In that study, the MD showed the highest activity during flexion, abduction, and internal rotation in patients after RSA at all analyzed time points (6 weeks, 3 months, and 6 months after surgery). It was also observed that during abduction, the MD cooperated with the AD, whereas during rotation it cooperated with the PD. These findings indicate that the MD is the principal contributor to movement in patients after RSA [19] (Table 2).

Similarly, in the study by Walker et al. (2014) [23], which also used sEMG, it was demonstrated that in patients after RTSA, AD activity was significantly higher during loaded abduction as well as during both unloaded and loaded flexion. However, in the case of the PD, the increase in activity was only minor [23] (Table 2).

In the study by Li et al. (2020) [21], D muscle activity was assessed preoperatively, and the obtained results were compared with postoperative outcomes. A positive correlation was demonstrated between preoperative D activity and postoperative shoulder girdle muscle strength after RSA. It was also observed that postoperative range of motion was positively correlated with the preoperative EMG values of the D muscle [21] (Table 2).

##### iEMG—Deltoid Muscle

In the study by Fischer et al. (2017) [16], iEMG was performed using disposable needle electrodes to assess the activity of the LD in patients after RSA in order to evaluate potential chronic neurogenic damage of the axillary nerve resulting from the procedure. The study demonstrated a probable absence of nerve injury, which was evidenced by the lack of correlation between either single motor unit potential (MUP) amplitude values or averaged MUP values for patients and the thickness of the examined muscle. Additionally, no correlation was observed between MUP amplitude and radiological or clinical outcomes [16] (Table 2).

##### sEMG—Trapezius Muscle

The second most frequently examined muscle was the T, particularly its upper portion (UT) [19,21,22,23]. In the study by Pietroski et al. (2025) [19], in which EMG assessments were performed at different postoperative time points after RSA, increased UT activation was demonstrated during abduction, forward flexion, and internal rotation (Table 2).

In the study by Pelletier-Roy et al. (2021) [22], where postoperative RSA patients were compared with a healthy control group, UT was identified as the primary muscle responsible for movement generation in all examined directions and exhibited the highest activity in the RSA group compared with the remaining muscles. Similarly, in the study by Walker et al. (2014) [23], postoperative RSA patients were compared with healthy controls, and UT activity was found to be significantly higher during loaded abduction as well as during both unloaded and loaded flexion. In contrast, in the study by Li et al. (2020) [21], which examined UT activity before surgery and compared it with postoperative outcomes, UT activation was shown to be positively correlated with postoperative shoulder girdle muscle strength (Table 2).

##### sEMG—Teres Minor Muscle

Two studies evaluated the activity of the TM [19,20]. In the study by Pietroski et al. (2025) [19], significantly increased TM activity was demonstrated during internal rotation 6 months after RSA. In contrast, the study by Rienmüller et al. (2020) [20] reported opposite findings, revealing a decrease in neuromuscular activation of the TM during external rotation (Table 2).

##### sEMG—Supraspinatus and Infraspinatus Muscles

One study observed an increase in SSP activation during internal rotation, which was observed 6 months after RSA. In contrast, no significant changes were observed for the IS muscle [19] (Table 2).

##### iEMG—Subscapularis Muscle

Only one study evaluated the SSC muscle using iEMG in patients after RSA. The examination was performed both before and after surgery and included the operated and non-operated sides. No changes were observed in EMG findings, and no evidence of muscle denervation as a result of RSA was detected in this study [51] (Table 2).

#### 3.2.2. ARCR—Arthroscopic Rotator Cuff Repair

In the study by Fritz et al. (2017) [31], iEMG was not performed either before or after ARCR; instead, the authors focused on assessing the effectiveness of rehabilitation. The study included 10 patients examined 9–12 weeks after surgical repair of torn rotator cuff tendons (RCT), and the results were compared with those of a healthy control group. Analysis of the iEMG data demonstrated a significant increase in SSC activity in patients after RCT repair during the performance of external rotation [31] (Table 2).

### 3.3. MyotonPRO

#### 3.3.1. Principles of MyotonPRO Operation

Measurement using the MyotonPRO device is non-invasive and rapid, lasting between 3 and 30 s. The procedure involves positioning the myometer probe perpendicularly to the skin surface at a precisely defined anatomical landmark. The examination is based on the activation of an electromagnetic mechanism that generates mechanical impulses of constant force through the probe, causing tissue deformation at the measurement site. The myometer automatically delivers a series of such impulses [35,52,53,54].

**Figure 3:**
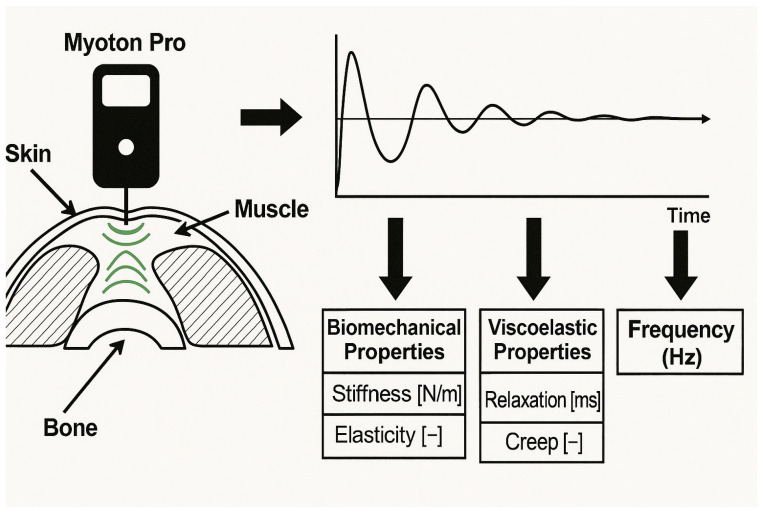
A graphical presentation of how MyotonPRO works [53,54].

Each probe impulse is brief (10–15 ms), of low force (0.40 N), and separated by a short inter-impulse interval (0.8 ms). No reflex neurological response of the examined tissue is elicited during the examination. The dynamic tissue response, consisting of displacement and oscillation, is recorded as a physical signal. Based on this signal, parameters characterizing the biomechanical and viscoelastic properties of the examined tissue are calculated, allowing functional assessment. MyotonPRO can be used to evaluate muscles, tendons, ligaments, skin, and fascia [35,53,54] (Figure 3).

##### Muscle Tone

Frequency—F [Hz]—This represents the natural oscillation frequency and characterizes the intracellular tension of the muscle. At high levels of intracellular muscle tension, blood flow within the muscle may become restricted. This results in faster muscle fatigue and delayed muscle recovery [35,53,54].

##### Biomechanical Properties

Stiffness—S [N/m]—This represents the resistance of the muscle to contraction or to an external deforming force and is calculated as the ratio of the force applied to the muscle (F) to the magnitude (depth) of tissue deformation (Δl). Greater muscle stiffness results in a more rapid rise in force. This is particularly important in certain sports disciplines, such as athletic throwing events (hammer, javelin, shot put). Greater stiffness and elasticity of a given muscle predispose it to rapid force development. In situations where a large joint range of motion is required, lower stiffness of the muscles surrounding the joint is desirable [35,53,54].

Decrement—D {-}—This parameter describes muscle elasticity and represents an index of oscillation damping resulting from mechanical energy dissipation. It reflects the muscle’s ability to return to its original shape after contraction or after removal of an external force. Proper elasticity ensures efficient utilization of muscle function [35,53,54].

##### Viscoelastic Properties

Relaxation—R [ms]—This is the time interval between the moment of maximum deformation of muscle tissue and its return to the initial state. In other words, relaxation defines how long the muscle needs to recover its original shape after voluntary contraction or after removal of an external force. It represents the time of mechanical stress relaxation in the muscle. Relaxation characterizes the recovery time of the tissue after deformation; the higher the tissue tension or stiffness, the faster the tissue regains its shape [35,53,54].

Creep—C—This parameter describes the creep property of the tissue and reflects the gradual elongation of muscle tissue over time under the influence of a stretching force. It is calculated as the ratio of the mechanical stress relaxation time (R) to the time required for tissue elongation (t_1_—tP). Here, tP denotes the baseline time point (zero deformation), and t_1_ represents the time of maximum elongation [35,53,54].

#### 3.3.2. Assessment of Rotator Cuff Muscles and Selected Muscles Important for Shoulder Joint Function Using MyotonPRO

##### Assessment of Upper Limb Muscles After ARCR/RSA

Only a very limited number of studies have investigated the use of MyotonPRO in patients after ARCR or RSA. We found only one article where MyotonPRO was just in patients after ARCR. This study evaluated the effect of radial extracorporeal shock wave therapy (rESWT) in patients who had undergone ARCR. In this work, the SSP and IS muscles were assessed. MyotonPRO measurements demonstrated an improvement in SSP tension following rESWT. However, the authors noted that the observed changes may have resulted from an increase in muscle thickness, rather than being a direct effect of the therapy itself [55]. Overall, direct evidence regarding MyotonPRO application after ARCR and RSA remains extremely limited (Table 3).

#### 3.3.3. Indirect Evidence: Feasibility and Reliability of MyotonPRO in Shoulder-Region Muscles

##### Evaluation of Muscles in Various Musculoskeletal Diseases

Table 3 summarizes two articles in which results were published concerning the assessment of upper limb muscle tissue quality in various diseases affecting the musculoskeletal system [56,57]. One study evaluated various neuromuscular diseases, including amyotrophic lateral sclerosis, spinal muscular atrophy, non-myotonic and myotonic myopathy, peripheral neuropathy, and myositis, and their effects on upper limb muscles [56]. Another study investigated the influence of Parkinson’s disease on the properties of upper limb muscles [57] (Table 4).

In the studies presented in Table 3, muscles were examined at rest [56,57]. The most frequently tested muscles using MyotonPRO were the PM [57] and the D [56] (Table 3).

In the study by Lukas et al. (2023) [56], it was observed that MyotonPRO did not produce fully reliable results. However, it should be emphasized that the study group included patients with five different conditions affecting both the musculoskeletal and nervous systems. The article did not specify the number of patients within each disease subgroup but only reported the total number of patients (n = 52), while the control group consisted of 21 individuals [56] (Table 4).

In contrast, the study by Zippenfening et al. (2023) [57] demonstrated the clinical value of this device. In this study, it was observed that the PM exhibited significantly higher frequency, stiffness, and decrement values in the morning before drug administration in patients with Parkinson’s disease compared with healthy controls [57] (Table 4).

##### Assessment of Upper Limb Muscles in Disease

Table 4 presents four publications in which the biomechanical properties of the following muscles were assessed: D, IS, UT, PM, and AD [58,59,60,61] (Table 5).

One study examined the effect of vitamin D deficiency on the stiffness, elasticity, and tension of selected upper limb muscles in patients aged 65–85 years. That study assessed the D muscle at rest and demonstrated that muscle elasticity measured using MyotonPRO depends on the patient’s serum vitamin D levels [58] (Table 5).

The remaining three studies evaluated the influence of chronic shoulder pain syndromes, asymmetry in rotational movements of the cervical spine, and adhesive capsulitis (AC) on the biomechanical properties of the examined muscles [59,60,61] (Table 5).

In the study by Roch et al. (2020) [59], which assessed the IS muscle at rest in patients suffering from chronic non-traumatic shoulder pain, it was demonstrated that MyotonPRO can significantly distinguish the viscoelastic properties of myofascial trigger points (TP) from non-trigger point (NTP) regions of the IS muscle (Table 5).

In the study by Wendt et al. (2024) [61], it was shown that asymmetry in cervical spine rotational movements was associated with an increase in stiffness of the UT on the side corresponding to the direction of asymmetry. Specifically, right-sided rotational asymmetry was associated with increased stiffness of the right UT, whereas left-sided asymmetry resulted in increased stiffness of the left UT [61] (Table 5).

Only in the study by Kurashina et al. (2023) [60] was the repeatability of MyotonPRO measurements formally assessed. This study confirmed that MyotonPRO results correlate with SWE measurements. Additionally, the authors observed that AD stiffness was higher on the side affected by adhesive capsulitis [60] (Table 5).

##### Assessment of Upper Limb Muscles After Another Surgical Procedures

Our review identified only one article that investigated the biomechanical properties of upper limb muscles using MyotonPRO following another surgical procedure than ARCR ora RSA [62] (Table 6).

This study focused on the PM and UT muscles in women who had undergone unilateral breast mastectomy and demonstrated that MyotonPRO is a reliable tool for assessing both stiffness and elasticity of the examined muscles. Moreover, a significant difference in the biomechanical properties of the PM muscle was observed when comparing the non-operated side with the operated side. Specifically, the healthy PM exhibited lower elasticity than the PM on the operated side [61] (Table 6).

##### Assessment of Upper Limb Muscles in a Healthy Population Using MyotonPRO

Our review identified six articles that examined the biomechanical properties of muscles surrounding the shoulder joint in a healthy population using MyotonPRO [63,64,65,66,67,68]. In three studies, the reliability and validity of the MyotonPRO device and the influence of testing conditions on measurement outcomes were evaluated [63,65,68]. One study investigated the effect of age on muscle biomechanical properties [64]. Two articles presented results on the impact of microgravity (absence of gravity) on muscle biomechanics [66,67] (Table 6).

In the studies presented in Table 6, muscles were most often examined at rest [65,66,67]. In two studies, the muscles were assessed both at rest and during contraction [59,64]. In one study, the biomechanical parameters of upper limb muscles were evaluated only during contraction [64] (Table 6).

The most frequently investigated muscles in the healthy population were the T and the D. The T was assessed in three studies [64,67], and the biomechanical properties of the D were also evaluated in three studies [65,66,67]. The IS muscle was examined in only one study [68] (Table 6).

Studies evaluating the reliability and validity of the MyotonPRO consistently demonstrated that it is a reliable tool for assessing the biomechanical properties of upper limb muscles [63,65,68]. A study assessing muscle biomechanical parameters in astronauts demonstrated that MyotonPRO can be used under different environmental conditions, including microgravity [66]. However, in the study by Amirova et al. (2021) [67], which also examined the effects of gravity, it was observed that muscle tension depended on the measurement site and varied according to study design [67]. In addition, the study evaluating MyotonPRO reliability during muscle contraction demonstrated that the results obtained during contraction are also reliable [68]. Finally, the study assessing the influence of age demonstrated that aging significantly affects biomechanical parameters such as stiffness and elasticity. Specifically, muscle stiffness increases with age, whereas elasticity decreases [64] (Table 7).

## 4. Discussion

Interest in shoulder joint surgery continues to increase, primarily due to the growing number of pathologies affecting this joint. This trend is not solely related to population aging and the development of degenerative joint changes but also to diseases involving the rotator cuff, including inflammatory tendon disorders as well as partial and complete tendon tears [69]. It should be emphasized that rotator cuff injuries are among the most common musculoskeletal conditions. Epidemiological studies have demonstrated that rotator cuff tendon tears may occur in more than 60% of the general population [70]. Furthermore, rotator cuff injuries have been identified as one of the most frequent causes of disability in adulthood. Surgical treatment of shoulder disorders is therefore widely applied, with ARCR and RSA being among the most frequently used procedures. Both surgical techniques aim to restore proper shoulder joint biomechanics and, consequently, its function [71].

In recent years, researchers have increasingly focused on changes in the biomechanics of the muscular system that occur as a result of muscle tissue degradation and synthesis associated with disease, injury, increased physical activity, immobilization, and aging [72]. Precise characterization of individual biomechanical properties of muscle tissue, such as stiffness and elasticity, enables monitoring of changes occurring within the muscle. Stiffness can be defined as the ability of a muscle to resist external forces and its reduced susceptibility to deformation during contraction [73]. The higher the stiffness, the greater the amount of energy required to change the shape of the muscle [74,75]. Elasticity, in turn, refers to the ability of muscle tissue to return to its original shape after contraction or after cessation of external forces. Numerous studies have demonstrated that quantitative assessment of stiffness and elasticity significantly contributes to understanding muscle function [73].

Injuries contribute to a loss of elasticity and an increase in stiffness. Studies have shown that immediately after injury, a reduction in elasticity occurs, followed by an increase in stiffness. If appropriate post-injury management is not implemented, this condition may progress to a chronic state [76,77].

The mechanical properties of muscles and tendons change not only following injury but also as a consequence of aging. Studies have demonstrated that muscle tissue stiffness increases with age. Sex also significantly influences muscle stiffness; however, these results are conflicting and dependent on the measurement method used. In a study by Wang et al. (2015) utilizing the free oscillation technique, men were found to exhibit greater muscle stiffness compared with women [78]. In contrast, a study by Eby et al. (2015) using elastography reported different findings [79]. It has also been demonstrated that strength training increases muscle stiffness [80]. Biomechanical properties of muscles also change in neurological disorders, including Parkinson’s disease and stroke [81]. Monitoring muscle status is also crucial in the postoperative period, as shown in studies evaluating the lumbar spine musculature [82].

Therefore, the aim of this review was to collect available evidence regarding whether ARCR and RSA lead to changes in the biomechanical properties of the rotator cuff and shoulder girdle muscles, and whether MyotonPRO has been applied in studies assessing these muscles following such surgical procedures.

The most commonly used methods for investigating muscle biomechanics include EMG and elastography [83]. Our team identified a total of 21 articles in which EMG or elastography was used to assess rotator cuff and/or shoulder girdle muscles in patients before and/or after ARCR or RSA [13,14,15,16,18,19,20,21,23,25,26,27,28,31,44,45,46,47,48,51].

The number of these articles indicates that the current literature on this topic contains limited research. It is also important to note that the number of patients participating in these studies is small, and the variability within the studied populations is very high (Table 1 and Table 2). This may be due to the fact that interest in shoulder joint surgery has recently been growing [69]. The limited number of studies as well as the variability of the studied populations may be influenced by the fact that study on how muscle biomechanical properties change in RSA and ARCR results has been conducted only recently have been conducted recently and require further research. Also, based on our review, it can be concluded that these studies are different in terms of methods of testing biomechanical properties. Elastography was applied in 13 studies [13,14,15,16,25,26,27,28,44,45,46,47,48], whereas EMG was used in 9 studies [16,18,19,20,21,22,23,31,51]. Interestingly, elastography was more frequently applied in patients evaluated before and/or after ARCR [25,26,27,28,45,46,47], whereas in patients examined before and/or after RSA, elastography was applied less frequently [13,14,15,16,44]. In contrast, EMG was used only once in patients after ARCR [31], while the remaining EMG studies focused on patients after RSA [16,18,19,20,21,22,23,51].

Our team identified 13 studies in which rotator cuff and/or shoulder girdle muscles were evaluated in patients after RSA [13,14,15,16,18,19,20,21,22,23,44,51]. Based on the collected literature, regardless of the biomechanical assessment method applied, the D muscle was the most frequently investigated muscle after RSA. Using elastography, the D was evaluated in five studies [13,14,15,44], whereas with EMG it was assessed in seven studies [16,18,19,20,21,22,23].

Elastography-based studies consistently demonstrated that RSA leads to significant changes in D biomechanics [13,14,15,16,44]. In most studies, the comparison was performed between the operated and non-operated sides [13,14,16]. Only the study by Dukan et al. (2022) [15] included a healthy control group in addition to bilateral comparisons. In the study by Hatta et al. (2024) [44], measurements were performed preoperatively and at 3, 6, 9, and 12 months postoperatively, with comparisons between the operated and non-operated sides.

Notably, regardless of the study design, all reviewed investigations demonstrated an increase in D stiffness and tension after RSA [13,14,15,16,44]. Additionally, Fischer et al. (2017) [16] reported a decrease in D elasticity. The observed increase in D stiffness after RSA is most likely related to postoperative healing and scar formation [84]. Furthermore, increased D stiffness is associated with changes in shoulder joint biomechanics following RSA, as this procedure leads to increased deltoid tension (DT) and activation [10].

It should be emphasized that relatively few studies have evaluated D stiffness longitudinally throughout the healing process after RSA. These studies are characterized by substantial heterogeneity of patient populations, different experimental protocols, and small sample sizes. The studies by Schmalzl et al. (2022) [13] and Fenwick et al. (2023) [14] demonstrated that increased D tension predominantly affects the anterior and middle portions of the muscle. Moreover, Schmalzl et al. (2022) [13] observed that increased anterior deltoid tension (ADT) correlated with increasing pain intensity [14].

In contrast, the elastography results reported by Fenwick et al. (2023) [14] were not correlated with functional shoulder assessment or radiographic evaluation of glenoid inclination or retroversion, which may be related to the limited sample size (n = 18) and high interindividual variability. Conversely, Hatta et al. (2024) [44] demonstrated a negative correlation between the increase in D stiffness measured at 3, 6, and 9 months postoperatively and the change in muscle strength observed three months later. The discrepancies between the findings of Hatta et al. (2024) [44] and Fenwick et al. (2023) [14] may be explained by differences in follow-up duration and study design. The follow-up period in the Fenwick et al. (2023) [14] study ranged from 4 to 48 months, whereas in the Hatta et al. (2024) [44] study patients were assessed every three months over a 12-month period and included a larger cohort (n = 65).

It should also be emphasized that in patients after RSA evaluated using elastography, only the D muscle was examined, most likely due to its superficial anatomical location and the technical ease of assessment [85]. RSA significantly alters shoulder joint biomechanics by modifying the anatomical configuration of the joint, thereby increasing the mechanical load on the D muscle. This surgical technique allows shoulder function to be restored with reduced reliance on the rotator cuff, leading to decreased pain and improved function in patients with advanced rotator cuff arthropathy [81,83,84]. However, as previously noted, RSA results in increased D activation, which may also contribute to D pathology [10]. This likely explains why the D muscle was so frequently and exclusively assessed in the reviewed studies [13,14,15,16,44].

In studies that assessed the D muscle in patients after RSA using EMG, partially divergent results were reported [16,18,19,20,21,22,23]. It should be noted that sEMG was used in six studies [18,19,20,21,22,23], whereas iEMG was employed in only one study [16]. The limited use of iEMG may be related to the fact that it is more invasive and time-consuming and requires considerable experience to ensure correct placement of needle electrodes [86,87,88]. However, the use of needle electrodes in that study made it possible to evaluate whether RSA leads to chronic neurogenic damage of the axillary nerve. It was shown that such damage was unlikely, as MUP values were not correlated with D thickness, nor were any correlations found between MUP amplitude and radiological or clinical outcomes [16]. It should also be emphasized that studies assessing complication rates after RSA have demonstrated that nerve injuries occur in approximately 1.3% of cases after primary RSA and in up to 2.4% of cases following revision RSA, with the axillary nerve being the most commonly affected (0.64%) [89]. These epidemiological data indicate that further research in this area is warranted.

In contrast, studies using sEMG have yielded inconsistent results. In the investigations by Pegreffi et al., 2017 [18], Rienmüller et al., 2020 [20], and Pelletier-Roy et al., 2021 [22], a reduction in D activity was observed. Both the studies by Pegreffi et al., 2017 [18] and Rienmüller et al., 2020 [20] included a two-year follow-up of patients after RSA and compared the operated to the non-operated side. Pegreffi et al. (2017) [18] reported lower AD and MD activity in all three analyzed movements on the RSA side. In the study by Reinmüller et al., 2020 [20], a reduction in LD activity on the operated side during flexion was observed, while PD activity was lower during extension and external rotation. In the study by Pelletier-Roy et al., 2021 [22], in which RSA patients were compared with healthy controls, AD activity during flexion was lower in the RSA group than in the control group.

On the other hand, Reinmüller et al., 2020 [20] also demonstrated increased antagonistic co-activation of AD during external rotation on the operated side compared with the non-operated side. Similarly, Pelletier-Roy et al., 2021 [22] observed a small increase in MD and PD activity during flexion and an increase in PD activity during external rotation in the RSA group. An increase in MD activity during flexion, abduction, and internal rotation at different postoperative time points (6 weeks, 3 months, and 6 months after RSA) was reported in the study by Pietroski et al., 2025 [19].

The discrepancies between the results reported in the studies included in this review may be attributed to differences in the number of enrolled patients, as well as to considerable variability in age, sex, and underlying pathology among the study populations [18,19,20,21,22,23]. It should also be noted that studies demonstrated that subcutaneous tissue thickness influences sEMG measurements [90]. To date, there are no studies directly comparing the effectiveness and utility of sEMG and iEMG in the assessment of patients after RSA.

Other muscles examined using EMG after RSA included the T [19,22,23], TM [19,20], SSP, IS [19], and SSC [51]. In EMG studies assessing the T, the UT was most frequently examined, and an increase in its activity after RSA was consistently reported [19,22,23]. In the study by Pietroski et al., 2025 [19], which compared pre- and postoperative results, UT activity increased during abduction, forward flexion, and internal rotation. In the studies by Pelletier-Roy et al. (2021) [22] and Walker et al. (2014) [23], RSA patients were compared with healthy controls. Pelletier-Roy et al. (2021) [22] showed that UT exhibited the highest activity among all assessed muscles in every tested direction (flexion, abduction, internal and external rotation). Walker et al. (2014) [23] reported increased UT activity during loaded abduction and during flexion with and without load. These findings indicate that UT plays a crucial role in movement execution in patients after RSA. This is consistent with its physiological function, as the T is essential for scapular stabilization and thus strongly influences shoulder joint biomechanics [4,91]. It has been demonstrated that RSA induces changes in shoulder joint biomechanics, leading to increased upward scapular rotation and enhanced activity of the scapulothoracic joint (STJ) [92], which may contribute to increased UT activation.

As mentioned earlier, the rotator cuff is composed of the TM, SSP, IS, and SSC muscles [1]. TM was evaluated in two studies in RSA patients. In the study by Pietroski et al. (2025) [19], increased TM activity during internal rotation was observed 6 months after surgery. Conversely, Rienmüller et al. (2020) [20] reported reduced TM activity during external rotation. These divergent findings may stem from the fact that they relate to different movements of the shoulder joint. Physiologically, TM is activated primarily during external rotation [93]. The observed decrease in TM activity during external rotation and concomitant increase during internal rotation may indicate that RSA induces changes in the biomechanics and function of this muscle, similar to what has been described for the T [92]. Although available data suggest that after RSA TM still contributes to external rotation of the shoulder joint [93], differences between the two studies may be partly explained by the lack of assessment of TM size and trophic index. Previous studies have shown that a trophic index below 0.75 may impair TM function in patients after such procedures [94]. It has also been demonstrated that muscle injury leads to structural changes, which can further affect its function [95]. It is noteworthy that in the study by Pietroski et al., 2025 [19], changes in TM activity were observed 6 months after RSA, whereas in Rienmüller et al., 2020 [20] they were recorded 2 years postoperatively, which may suggest progressive atrophy of this muscle.

Using sEMG, only one study evaluated both SSP and IS [44], while Khazzam et al. (2020) [51] assessed SSC using iEMG. These studies demonstrated changes only in SSP activity, with increased SSP activation observed during internal rotation 6 months after RSA [19]. The limited number of studies assessing SSC, IS, and SSP activity after RSA may be related to the anatomical location of these muscles, which makes them more difficult to evaluate using EMG [85]. Epidemiological data indicate that 40–70% of patients with shoulder pain have rotator cuff tears, most commonly involving the SST (61.9% of men and 38.1% of women) [24]. The second most frequently affected muscle is IS. In the case of SSC, 27.4% of rotator cuff tears involve this muscle; however, isolated SSC tears are rare and account for only 6–10% of all rotator cuff tendon lesions [96]. Based on our review, it can be concluded that the number of studies assessing SSC, IS, and SSP activity using EMG in RSA patients remains limited, highlighting an important direction for future research.

Our team identified a total of nine studies in which patients after ARCR were assessed using elastography or EMG [25,26,27,28,31,45,46,47,48]. Elastography was used in eight studies [25,26,27,28,31,45,46,47,48], while EMG was used in a single study [31].

In the ARCR population, elastography was applied to evaluate both muscles [25,26,27,28,31,45,46,47,48] and tendons [25,27,45,46,47]. The most frequently examined muscle and tendon after ARCR were SSP and SST [25,26,27,28,45,46,47,48]. SSP was assessed in five studies [26,27,28,45,48], as was SST [25,27,45,46,47]. The large number of studies focusing on this muscle–tendon unit may be explained by the fact that in patients with rotator cuff tears, the SSP insertion is most commonly involved [24]. It should also be emphasized that only the SST was evaluated; no other rotator cuff tendon was examined, likely due to their deeper anatomical location and greater technical difficulty of assessment [85].

In the study by Hackett et al. (2023) [46], no changes in SST stiffness were observed during the first 12 weeks after ARCR. However, at 6 months postoperatively, an increase in stiffness of the lateral portion and the medial portion 3 mm from the repair site was demonstrated. For the medial portion located 6 mm from the repair site, stiffness increased 6 weeks later [46]. An increase in SST stiffness at 6 and 12 months after ARCR was also reported by Solari et al. (2024) [47], who additionally demonstrated a positive correlation between SST thickness and tendon stiffness.

In contrast, Itoigawa et al. (2020) [27] examined whether SST stiffness after ARCR depends on the initial tear severity. They showed that in patients with small or partial tears, tendon stiffness was higher at 1 week postoperatively compared with 3, 4, and 6 months after surgery. In patients with medium and large tears, SST stiffness at 1 week was higher only compared with 3 and 6 months postoperatively [27]. These data indicate that SST stiffness changes over time during the healing process after ARCR and is influenced both by rotator cuff tear severity and by the exact measurement location [27,46,47]. Stiffness is also dependent on tendon thickness [47]. Similarly, Kim et al. (2023) [25] demonstrated that patients with stiffer SST after ARCR showed greater muscle strength compared with those with less stiff tendons. This suggests that increased tendon stiffness in the postoperative period may reflect better structural healing. Previous work has shown that stiffer tendons tend to be thicker [97], which was also observed by Solari et al. (2024) [47] in their ARCR cohort. Tendon thickening is associated with increased collagen fiber content, denser packing, and larger fiber diameter [97].

In the study by Chen et al. (2024) [45], the authors investigated whether preoperative SST stiffness is associated with ARCR outcomes. They observed that SWV values for SST were negatively correlated with postoperative results [45], indicating that higher preoperative SWV is associated with worse ARCR outcomes. As mentioned earlier, injury initially leads to a reduction in elasticity followed by an increase in stiffness [76,77]. However, it should be noted that higher tendon stiffness after ARCR may be associated with better healing and a lower retear rate at 6 months postoperatively [98].

In ARCR patients, several studies analyzed SSP to determine how activity in individual regions of the muscle changes in response to rotator cuff tears [26]. Investigators also examined whether preoperative SSP stiffness and elasticity correlate with ARCR outcomes [28,45,48], and whether tear severity affects SSP stiffness [28]. Additionally, changes in SSP stiffness during the healing process after ARCR were evaluated [27].

In the study by Sakaki et al. (2022) [26], SSP was divided into four regions based on muscle fiber orientation (PS, PD, AS, and AD). In patients with rotator cuff tears, active stiffness in the AS region was the lowest, suggesting that each SSP subregion contributes differently to shoulder function in this patient group. Several studies also demonstrated that SSP stiffness is associated with ARCR outcomes [26]. Jeong et al. (2022) [48] reported that higher preoperative SSP stiffness was observed in patients with insufficient rotator cuff repair. They also showed that elasticity may serve as a diagnostic marker to identify patients for whom ARCR alone may be insufficient and who might benefit from alternative treatment strategies [48]. Likewise, Chen et al. (2024) [45] found that preoperative SSP stiffness was negatively correlated with postoperative outcomes. In the study by Huang et al. (2022) [28], the authors assessed the influence of rotator cuff tear severity on SSP and IS stiffness and demonstrated a significant correlation between stiffness and both the severity and size of the tear. Similarly, Itoigawa et al. (2020) [27] showed that SSP stiffness depends on tear grade and changes over time during healing. These findings support the concept that stiffness influences the healing process and suggest that restoration of function and successful rotator cuff repair may be more difficult in patients with high preoperative muscle stiffness. Increased rotator cuff muscle stiffness may therefore indicate irreversible damage, prompting the surgeon to consider alternative procedures rather than standard ARCR [28,45,48]. Moreover, temporal changes in stiffness during healing may serve as a diagnostic marker for predicting the risk of retear [27].

In patients after ARCR, the D muscle has also been studied. Our team found two studies assessing D stiffness after ARC. Both demonstrated that D stiffness changes over time and is significantly higher 12 months after surgery [46,47]. These post-ARCR changes in D stiffness suggest that by 12 months after surgery, patients may be able to return to normal activity levels [47].

Of particular interest, our team identified only one study in which EMG was performed in patients after ARCR. Importantly, this study did not examine patients before and after surgery but focused on the rehabilitation period, evaluating the effectiveness of the rehabilitation program in this population. The sample size was small, including 20 individuals: 10 healthy controls and 10 patients who had undergone ARCR 9–12 weeks prior to study entry. iEMG analysis demonstrated a significant increase in SSC activity in the ARCR group during external rotation [31]. This is the only available study reporting such changes in SSC activity after ARCR. Physiologically, SSC is responsible for shoulder joint stability and is primarily active during internal rotation. After ARCR, SSC continues to contribute to internal rotation [99]. The absence of differences between the ARCR and control groups during internal rotation in the study by Fritz et al. (2017) [31] may simply reflect the fact that SSC is similarly active during this movement in both healthy individuals and ARCR patients. As noted above, to the best of our knowledge, this is the only study in which SSC activity was evaluated using iEMG and in which increased SSC activity during external rotation was demonstrated. There are currently no studies that have examined this muscle using sEMG, likely due to its anatomical location and the difficulty of reliable assessment [85]. Therefore, further research is recommended to determine whether EMG is a reliable tool for assessing SSC activity in patients after ARCR.

A unique contribution of our review is the observation that no studies have evaluated rotator cuff or shoulder girdle muscles in patients before or after RSA using MyotonPRO. Furthermore, only one study has assessed the biomechanical and viscoelastic properties of SSP and IS in patients after ARCR using MyotonPRO [55]. Our team identified two studies in which the D muscle [56] and PM [57] were examined in patients with neuromuscular or musculoskeletal conditions such as non-myotonic and myotonic myotonias, amyotrophic lateral sclerosis, spinal muscular atrophy, peripheral neuropathy, myositis [56], and Parkinson’s disease [57]. We also found four studies in which D [58], IS [59], UT [61], PM and AD [60] were assessed in the context of specific conditions, including hypovitaminosis D3 [58], chronic shoulder pain syndromes [59], asymmetry in cervical spine rotational movements [61], and AC [60]. In one study, changes in PM and UT properties were evaluated in women after a mastectomy [62]. Finally, our literature review identified six articles in which the properties of rotator cuff muscles (IS) [68] and shoulder girdle muscles (T and D) [63,64,65,66,67] were examined in healthy populations using MyotonPRO.

Five studies showed that MyotonPRO is a reliable tool for assessing the properties of shoulder girdle and rotator cuff muscles [60,62,63,64,65]. Other studies, which were not included in this review due to differences in keywords, likewise confirmed that MyotonPRO is a valid instrument for evaluating upper limb muscles [36,37,39,100,101,102]. Furthermore, in the study by Schoenrock et al., 2024 [66] involving astronauts, it was demonstrated that MyotonPRO can be used under microgravity conditions, indicating that measurements can be reliably performed in virtually any environment.

Only the study by Lukas et al. (2023) [56] did not confirm the reliability of the device. In that study, the patient group consisted of 52 individuals and the control group of 21 healthy volunteers. The patient cohort included individuals with a range of neuromuscular and musculoskeletal conditions affecting both the muscular and nervous systems, such as amyotrophic lateral sclerosis, spinal muscular atrophy, non-myotonic and myotonic myopathy, peripheral neuropathy, and myositis. However, the number of patients in each diagnostic subgroup was not specified, which may have substantially affected the reliability of the results [56]. Similarly, in the study by Amirova et al. (2021) [67], which evaluated the effects of gravity, it was observed that muscle tone depended on the measurement site and varied according to study design. These discrepancies indicate that further research is required in this area.

In studies conducted in patients with disorders affecting the musculoskeletal system, as well as in other conditions influencing muscle tissue, MyotonPRO measurements have been shown to detect disease-related changes in muscle properties [57,58,59,60,61].

In addition, one of the studies included in our review examined the effect of age on muscle stiffness. That study demonstrated that stiffness increases with age, whereas elasticity decreases [64]. Other studies not included in this review also showed that age, sex, physical activity, the specific muscle examined, and contraction state may influence MyotonPRO results [32,35,100,101,103,104,105]. These factors affect parameters such as stiffness, elasticity, and muscle tone. The influence of age, sex, and activity level on muscle biomechanical properties has also been confirmed in studies using other measurement techniques, as discussed earlier [72].

In the only study to date that evaluated patients after ARCR using MyotonPRO during rehabilitation—performed 6 weeks after surgery—it was shown that rESWT improved SSP tone [55]. However, there are no published studies in which MyotonPRO has been used to monitor how the biomechanical and viscoelastic properties of rotator cuff and shoulder girdle muscles change over time as a result of ARCR or RSA. Likewise, there is a lack of studies assessing the reliability of MyotonPRO in this context and comparing its results directly with currently used techniques such as EMG or elastography.

Further research in this field will help clarify how muscle tissue changes after ARCR and RSA, thereby supporting the selection of optimal treatment strategies and postoperative rehabilitation protocols to facilitate the fastest possible recovery. The limited number of studies using MyotonPRO in these patient groups, as observed in our review, indicates a clear opportunity for further investigation. This is particularly relevant given that the device is easy to use, measurements can be performed in diverse settings [36,66], and numerous studies in patients with different muscle-related conditions—as well as in healthy populations—have shown that MyotonPRO is robust and yields reliable results [36,37,38,60,62,63,65,66,68,100,101,102,106]. It should nevertheless be emphasized that several studies have reported that sex, age, physical activity, and contraction state may influence MyotonPRO-derived measurements [32,35,64,100,101,103,105], while others did not confirm these effects [68,104,105]. For this reason, we recommend conducting studies in highly homogeneous patient groups, which will improve the reliability and interpretability of the results [34]. In terms of clinical relevance, our team’s literature review demonstrates the importance of muscle biomechanical properties before and after ARCR and RSA. RSA contributes to changes in the anatomical structure of the shoulder joint, which may influence changes in the biomechanical properties of the muscles within this joint, as demonstrated specifically by changes in the activity and function of the D and T muscles. This method allows for changes in the biomechanics of the rotator cuff without requiring their use, which contributes to a reduction in pain perception and thus improves function in patients with advanced rotator cuff arthropathy [10,11,12]. In the studies discussed in our work, it can be seen that the D and T muscles are very important during movement in patients after RSA. These studies may indicate that new rehabilitation protocols for patients after RSA should be developed, taking into account the changes in muscle biomechanics that occur as a result of this surgical technique. However, it should be noted that the current number of studies dealing with the topic of changes in muscle biomechanics in RSA results is organic and the variability of the subjects is small, so further research in this direction should be conducted [13,14,15,16,18,19,20,21,22,23,44,51]. In patients who have undergone ARCR, clinical evidence has shown that the stiffness of the repaired SST changes during the healing process. It has also been observed that not only does tendon stiffness change, but also that preoperative muscle stiffness itself influences the healing process. This may indicate that restoring function and correcting rotator cuff repair may be difficult in patients with high preoperative muscle stiffness. This study also shows that increased rotator cuff muscle stiffness should suggest that the damage is permanent and irreversible, and in this case, the surgeon should use a surgical method other than ARCR [28,45,48]; whereas changes in stiffness during healing may be a diagnostic factor in patients that may be important in predicting recurrent rotator cuff tears [27]. From a clinical perspective, MyotonPRO may be a suitable tool for examining changes in muscle biomechanical properties in patients before and after ARCR and RSA. This device can be used as a diagnostic tool because it is not expensive and is practical and non-invasive. Also, this devise does not require specialized laboratory conditions, and the examination is easy to perform, requiring only appropriate training and the selection of correct anatomical landmarks for measurements [36,37,38,60,62,63,65,66,68,100,101,102,106]. However, based on the literature collected by our team, it can be seen that the number of studies using this device is small; therefore, further research in this direction is necessary.

The main limitation of our review is the absence of a meta-analysis of the included publications. The next limitation of our paper is the lack of use of electronic tools for reviewing the literature. Another limitation is that the included studies were characterized by relatively small sample sizes, heterogeneous patient populations, variability in surgical techniques and rehabilitation protocols, and differences in biomechanical assessment methods. Moreover, another limitation of our work in the case of the described studies and patient groups is the lack of analysis of confounding factors such as comorbidities, physical activity and postoperative rehabilitation time. These factors limit the comparability of findings and preclude firm clinical conclusions. However, regardless of these limitations, our team pointed out that research on this topic is limited. Nevertheless, we observed a trend that the biomechanical properties of the rotator cuff muscles and shoulder girdle may change as a result of ARCR and RSA. We also demonstrated that is a significant lack of clinical, case–control, and randomized studies using MyotonPRO to assess the biomechanical and viscoelastic properties of muscles surrounding the shoulder joint in patients undergoing ARCR and RSA. This gap highlights a clear and clinically relevant direction for future research.

## 5. Conclusions

This scoping review demonstrates that surgical procedures such as arthroscopic rotator cuff repair (ARCR) and reverse shoulder arthroplasty (RSA) are associated with measurable changes in the biomechanical properties of shoulder-region muscles, as assessed primarily by elastography and electromyography.

However, direct evidence regarding the use of MyotonPRO in patients after ARCR and RSA remains extremely limited. Only isolated studies have applied this device in postoperative shoulder populations, and no robust longitudinal trials specifically addressing postoperative biomechanical adaptation using MyotonPRO are currently available.

Indirect evidence from studies conducted in other clinical contexts suggests that MyotonPRO is a technically feasible and reliable tool for assessing biomechanical and viscoelastic muscle properties in shoulder-region musculature. Nevertheless, these findings cannot be directly extrapolated to postoperative ARCR/RSA populations.

Given the heterogeneity of existing studies, small sample sizes, and variability in assessment protocols, the current evidence should be interpreted as exploratory. Well-designed prospective studies with standardized measurement protocols are required before MyotonPRO can be recommended for routine clinical implementation in patients undergoing ARCR or RSA.

Future research integrating MyotonPRO with imaging and functional outcome measures may contribute to a more comprehensive understanding of postoperative muscular adaptation following shoulder reconstructive procedures.

## Figures and Tables

**Table 1:** Use of elastography in the assessment of muscles surrounding the shoulder joint in patients after RSA and ARCR.

Authors	Patients	Muscles/Tendons	Main Findings
RSA			
Schmalzl et al., 2022 [13]	N = 18; Operated vs. non-operated; At rest.	AD; MD; PD.	Changes in biomechanical properties in AD and MD. Increased tension in AD correlated with higher pain (VAS).
Fenwick et al., 2023 [14]	N = 18; Operated vs. non-operated; At rest.	AD; MD; PD.	Increased tension in AD and MD after RSA vs. contralateral side. No correlation with function due to high variability.
Dukan et al., 2022 [15]	Control N = 26; RSA N = 12 (Operated vs. non-operated); At rest and abduction	AD; MD; PD.	Significant resting stiffness increased in AD and MD vs. controls. No differences during abduction.
Fischer et al., 2017 [16]	N = 65; Operated vs. non-operated; At rest.	D	Greater stiffness and lower elasticity of D on the operated side (ARFI-based SWE).
Hatta et al., 2024 [44]	N = 65; Pre-op and 3, 6, 9, 12 months post-op.	D (5 regions: 2 AD, 1 MD, 2 PD).	Higher postoperative stiffness negatively correlated with later muscle strength improvement.
ARCR			
Kim et al., 2023 [25]	N = 42; 3 and 6 months post-op.	SST	Higher SST stiffness associated with better strength and function recovery.
Sakaki et al., 2022 [26]	Control N = 13; ARCR N = 13 (Pre-op); During abduction.	SSP (PS, PD, AS, AD)	Reduced stiffness of AS SSP region in rotator cuff tears.
Itoigawa et al., 2020 [27]	N = 60; Pre-op and up to 6 months follow-up.	SST; SSP (PS, PD, AS, AD).	Dynamic stiffness changes during healing; stiffness depended on tear severity.
Huanga et al., 2022 [28]	Operated N = 97; Control N = 36; At rest.	SSP; IS.	Muscle stiffness correlated with tear size and severity.
Chen et al., 2024 [45]	Operated N = 89; Contralateral N = 40; Control N = 10.	SSP (PS, PD, AS, AD); SST (TPS, TPD, TDS, TDD).	Lower SWV in operated shoulders; pre-op stiffness negatively correlated with outcomes.
Hackett et al., 2023 [46]	N = 50; 1–52 weeks follow-up; At rest.	SST (3 regions); D.	D stiffness increased by 15% by 52 weeks. SST stiffness increased by 21% at 6 months.
Solari et al., 2024 [47]	N = 48; 1 week–12 months follow-up; At rest.	SST; D.	Significant D and SST stiffness increases at late follow-up; positive SSP–SST correlation.
Jeong et al., 2022 [48]	N = 74; Pre-op; At rest.	SSP	Higher pre-op stiffness associated with insufficient repair; SWE complementary to MRI.

Legend: N—sample size; SWV—shear wave velocity; SWE—shear wave elastography; ARCR—arthroscopic rotator cuff repair; RSA—reverse shoulder arthroplasty; D—deltoid; AD—anterior deltoid; MD—middle deltoid; PD—posterior deltoid; SST—supraspinatus tendon; SSP—supraspinatus muscle; IS—infraspinatus muscle; Pre-op—preoperative.

**Table 2:** Use of EMG in the assessment of muscles surrounding the shoulder joint in patients after RSA and ARCR.

Authors	Patients	sEMG/iEMG	Muscles	Main Findings
RSA				
Pegreffi et al., 2017 [18]	N = 20; Operated vs. non-operated side; Flexion, abduction, extension.	sEMG	AD; MD; PD.	Overall lower muscle activity on the operated side in all movements. Specifically, reduced AD and MD activity compared with the non-operated side at 2 years after RSA.
Fischer et al., 2017 [16]	N = 64; At rest and during contraction.	iEMG	LD	No association between MUP amplitude and muscle thickness. No correlation with radiological or clinical outcomes, indicating no neurogenic injury.
Khazzam et al., 2020 [51]	N = 20; Pre- and postoperative; Operated and non-operated side; At rest.	iEMG	SSC	Preserved SSC function after RSA. No signs of SSC denervation.
Pietroski et al., 2025 [19]	N = 10; Pre-op and 6 weeks, 3 and 6 months post-op; During contraction.	sEMG	AD; MD; PD; SSP; IS; TM; UT; LT.	Greatest increase in MD activity during flexion, abduction and rotation. Increased UT activity. Higher SSP and TM activity during internal rotation. MD identified as primary mover after RSA.
Rienmüller et al., 2020 [20]	N = 13; Operated vs. non-operated; 2 years post-RSA; Various movements.	sEMG	AD, MD, LD, PD, TM.	Reduced LD activity during flexion on the operated side. Reduced PD activity during extension and external rotation. Reduced AD activity during internal rotation. Increased AD co-activation during external rotation. Decreased TM activity during external rotation.
Li et al., 2020 [21]	N = 25; Pre-operative; Shrugging, forward flexion, abduction.	sEMG	D; UT.	D and UT activity positively correlated with postoperative shoulder strength and range of motion after RSA.
Pelletier-Roy et al., 2021 [22]	RSA N = 11; Controls N = 10; Various shoulder movements.	sEMG	UT; AD; MD; PD.	UT was the main activator in all movements. AD activity was lower during flexion vs. controls. Slight increase in MD and PD activity. PD activity higher during external rotation.
Walker et al., 2014 [23]	RSA N = 33; Controls N = 17; Flexion, external rotation, abduction with and without load.	sEMG	AD; MD; LD; PD; UT.	Higher activity of all examined muscles in RSA patients. AD and UT showed significantly higher activation during loaded abduction and flexion.
ARCR				
Fritz et al., 2017 [31]	ARCR N = 10; Controls N = 10; 9–12 weeks post-op; During contraction.	iEMG	SSC	Significant increase in SSC activity during external rotation after ARCR.

Legend: N—sample size; iEMG—intramuscular electromyography; sEMG—surface electromyography; ARCR—arthroscopic rotator cuff repair; RSA—reverse shoulder arthroplasty; D—deltoid; AD—anterior deltoid; MD—middle deltoid; PD—posterior deltoid; LD—lateral deltoid; SSP—supraspinatus; IS—infraspinatus; UT—upper trapezius; LT—lower trapezius; TM—teres minor; SSC—subscapularis.

**Table 3:** Use of MyotonPRO in the assessment of skeletal muscle quality of the upper limb after ARCR/RSA.

References	Study Design	Type of Surgical Procedure	Muscles	Conclusions
Kim et al., 2022 [55]	N = 30 (16M/14F); Average age: 49.47 ± 5.22 years; Before and after rESWT.	ARCR	SSP; IS.	The MyotonPRO assessment demonstrated an improvement in SSP tension after rESWT, which may also be related to increased muscle thickness.

Legend: N—number of participants; F—female; M—male; SSP—supraspinatus; IS—infraspinatus; rESWT—radial extracorporeal shock wave therapy; ARCR—arthroscopic rotator cuff repair.

**Table 4:** Use of MyotonPRO in the assessment of skeletal muscle and tendon quality of the upper limb in diseases affecting muscle function.

Authors	Study Design	Disease	Muscle	Conclusions
Lukas et al., 2023 [56]	N = 73 total; N = 52 patients with neuromuscular diseases; N = 21 healthy controls.	ALS, spinal muscular atrophy, non-myotonic and myotonic myotonias, peripheral neuropathy, myositis.	D; At rest.	MyotonPRO was unable to reliably detect changes in measured values or provide clear indicators of underlying muscle disease.
Zippenfening et al., 2023 [57]	N = 91; N = 49 patients with Parkinson’s disease (24M/25F, mean age 69.76 ± 6.39 years); N = 42 healthy controls (21M/21F, mean age 60.48 ± 7.62 years).	Parkinson’s disease	PM; At rest.	PM showed significantly higher frequency, stiffness, and decrement in the morning before medication intake compared with healthy controls.

Legend: N—sample size; F—female; M—male; D—deltoid; PM—pectoralis major.

**Table 5:** Use of MyotonPRO in the assessment of skeletal muscle quality of the upper limb in disease.

References	Study Design	Disease	Muscles	Conclusions
Kocaer et al., 2021 [58]	N = 109 (21M/88F); patients divided into three groups based on serum vitamin D levels: sufficient (≥30 ng/mL), insufficient (21–29 ng/mL), and deficient (<20 ng/mL); mean age: 71.2 years (65–85 years).	Hypovitaminosis D3	D; At rest.	Muscle strength, flexibility, and physical fitness were associated with vitamin D levels. Proximal muscle strength measured with a handheld dynamometer may predict hypovitaminosis D in older adults.
Roch et al., 2020 [59]	N = 35 (23F/12M); mean age: 42 years; non-traumatic chronic shoulder pain ≥2/10 (NRS) lasting > 3 months; BMI < 28.	Chronic shoulder pain syndromes	IS; At rest.	MyotonPRO differentiated the viscoelastic properties of trigger points (TP) from non-trigger points (NTP) in the infraspinatus muscle.
Wendt et al., 2024 [61]	N = 60 (36M/24F); mean age: 20 years; N = 22 experimental group with cervical rotation asymmetry; N = 38 control group.	Asymmetry in rotational movements in the cervical spine	UT; At rest.	Increased stiffness of the right UT was observed with right-sided asymmetry. Left-sided asymmetry was associated with increased stiffness of the left UT.
Kurashina et al., 2023 [60]	N = 50; N = 40 patients with adhesive capsulitis; N = 10 control group.	Adhesive capsulitis (AC)	PM; AD; At rest.	MyotonPRO results were repeatable and correlated with SWE findings. LD and AD stiffness were higher on the AC-affected side.

Legend: N—number of subjects; F—female; M—male; D—deltoid; IS—infraspinatus; TP—trigger point; NTP—non-trigger point; UT—upper trapezius; PM—pectoralis major; AD—anterior deltoid; SWE—shear wave elastography.

**Table 6:** Use of MyotonPRO in the assessment of skeletal muscle quality of the upper limb after surgical treatment.

References	Study Design	Type of Surgical Procedure	Muscles	Conclusions
Yeo et al., 2019 [62]	N = 22F; Average age: 41.1 years (30–70 years); After unilateral mastectomy due to breast cancer; After chemotherapy and radiotherapy; Time from surgery to examination: 29 months.	Mastectomy	PM; TU.	MyotonPRO is a reliable tool for assessing the properties of the PM and TU muscles (stiffness, tone and elasticity) in post-mastectomy patients. A significant difference in elasticity was observed only for the PM muscle, with lower elasticity on the unoperated side.

Legend: N—number of participants; F—female; PM—pectoralis major; TU—upper trapezius.

**Table 7:** Use of MyotonPRO in the assessment of skeletal muscle and tendon quality of the upper limb in a healthy population.

References	Study Design	Muscles	Conclusions
Liu et al., 2018 [63]	N = 20M; mean age: 28.3 years; mean height: 172 cm; mean weight: 66.7 kg; individuals with neck pain, instability, fractures, upper limb surgeries, or using muscle relaxants/steroids were excluded.	T; At contraction and rest.	Reliability confirmed. Trapezius stiffness increased during flexion from 0° to 60°.
Kocur et al., 2019 [64]	N = 95F; mean age: 48.8 years; BMI: 24.6; only women without neck and cervical spine pain in the previous 6 months.	T; At contraction.	Muscle stiffness increased and elasticity decreased with age.
Muckelt et al., 2022 [65]	N = 20 (10F/10M); mean age: 28.95 years; BMI: 24.28.	D; At rest.	MyotonPRO measurements showed high reliability for tension, elasticity, and stiffness.
Schoenrock et al., 2024 [66]	N = 12 astronauts (4F/8M) during long-term missions.	D; At rest.	MyotonPRO is usable in various conditions. Muscle stiffness may serve as a digital biomarker for health monitoring in space.
Amirova et al., 2021 [67]	N = 12 (6F/6M); mean age: 32.8 years; healthy volunteers with normal physical activity and no musculoskeletal disorders.	D; T; At rest.	Muscle tension depended on test site and study design. A standardized testing protocol is recommended.
Kelly et al., 2018 [68]	N = 30 (13F/17M); mean age: 27.87 years; BMI < 30; healthy volunteers.	IS; At contraction and rest.	MyotonPRO is reliable in both contracted and resting muscle states.

Legend: N—number of participants; F—female; M—male; T—trapezius; D—deltoid; IS—infraspinatus; BMI—body mass index.

## Data Availability

Data are contained within the article.

## Supplementary Materials

The following supporting information can be downloaded at: https://www.mdpi.com/article/10.3390/jcm15052039/s1, Supplementary File S1: PRISMA-ScR checklist [107].

## Abbreviations

The following abbreviations are used in this manuscript:

AD	anterior deltoid
ARCR	arthroscopic rotator cuff repair
BMI	body mass index
D	deltoid
EMG	electromyography
F	female
iEMG	intramuscular electromyography
IS	infraspinatus muscle
LD	lateral deltoid
LT	lower trapezius
M	male
MD	middle deltoid
MT	middle trapezius
N	sample size
NTP	non-trigger point
PD	posterior deltoid
PM	pectoralis major
Pre-op	preoperative
rESWT	radial extracorporeal shock wave therapy
RSA	reverse shoulder arthroplasty
SE	static elastography
sEMG	surface electromyography
SSC	subscapularis
SSP	supraspinatus muscle
SST	supraspinatus tendon
SWE	shear wave elastography
SWS	shear wave speed
SWV	shear wave velocity
TM	teres minor
TP	trigger point
UT	upper trapezius
